# Influence of Coffee Roasting Degree from Four Mexican Regions on In Vitro Antioxidant Activity and Digestive Enzyme Inhibition and Its In Vivo Effects on Carbohydrate and Lipid Absorption

**DOI:** 10.3390/ijms262010067

**Published:** 2025-10-16

**Authors:** Claudia I. Gamboa-Gómez, Jazel Barragán-Zúñiga, Mayra Denise Herrera, Marilisa Alongi, Nuria E. Rocha-Guzmán, Karen M. Herrera-Rocha, Deisy Dominguez, Karla F. Valles-Araiza, Monica Anese, Martha Rodríguez-Morán, Fernando Guerrero-Romero

**Affiliations:** 1 Unidad de Investigación Biomédica del Instituto Mexicano del Seguro Social, Canoas 100, Durango 34067, Mexico; qfb.deisydv.2309@gmail.com (D.D.); fervaqfb@gmail.com (K.F.V.-A.); rodriguez.moran.martha@gmail.com (M.R.-M.); 2 Centro Estatal de Cancerología, Secretaria de Salud Durango, Av. 5 de Febrero esq. Antonio Norman Fuentes S/N Zona Centro, Durango 34000, Mexico; ljbarraganz@gmail.com; 3 Instituto Nacional de Investigaciones Forestales Agrícolas y Pecuarias, Campo Experimental Zacatecas Kilómetro 24.5, Fresnillo 98500, Mexico; mayradherrera@gmail.com; 4 Department of Agricultural, Food, Environmental and Animal Sciences, University of Udine, Via Sondrio 2/A, 33100 Udine, Italy; marilisa.alongi@uniud.it (M.A.); monica.anese@uniud.it (M.A.); 5 TecNM/Instituto Tecnológico de Durango/Laboratorio Nacional Conahcyt de Apoyo a la Evaluación de Productos Bioticos (LaNAEPBi), Blvd. Felipe Pescador 1830 Ote, Colonia Nueva Vizcaya, Durango 34080, Mexico; nrocha@itdurango.edu.mx (N.E.R.-G.); kherrera@itdurango.edu.mx (K.M.H.-R.)

**Keywords:** Mexican coffee, roasting grade, phytochemical profile, in vitro and in vivo activity

## Abstract

Coffee is among the most consumed beverages worldwide and is recognized for its bioactive compounds, which exert diverse physiological effects. This study evaluated the impact of roasting degree on the in vitro antioxidant activity and digestive enzyme inhibition of brews from four Mexican regions, as well as their in vivo effects on carbohydrate and lipid absorption. Antioxidant capacity was assessed through radical scavenging and lipid peroxidation inhibition, while inhibition of lipase, α-amylase, and α-glucosidase was also determined. Oral starch (OSTT) and lipid (OLTT) tolerance tests were conducted in healthy Wistar rats. Antioxidant activity was strongly influenced by region and roasting degree. All coffee samples exhibited radical scavenging activity and lipid peroxidation inhibition. With respect to enzyme inhibition, all coffees showed ~67–70% inhibition of lipase activity. For amylase, unroasted coffee from Oaxaca displayed the highest inhibition (34%, *p* < 0.001). For glucosidase, unroasted samples showed low inhibition (~6–19%), which increased substantially at the medium roast degree (~55% across all samples) but decreased again at the high roast degree (~27%). In OSTT, serum glucose levels were reduced after 120 min by ~20%, 21%, and 18% in rats treated with unroasted, medium-roast, and high-roast coffee, respectively, compared with the negative control. In OLTT, serum triglycerides decreased by ~26% (Chiapas), ~58% (Colima), ~32% (Oaxaca), and ~54% (Hidalgo). Crop region and roasting degree influence the phytochemical profile and bioactivity of Mexican coffee. Although unroasted coffees had the highest concentration of bioactive compounds, roasting enhanced specific bioactivities, particularly enzyme inhibition and lipid-lowering effects in vivo.

## 1. Introduction

Coffee is among the most consumed beverages worldwide and is recognized for its bioactive compounds, which exert diverse physiological effects. Its global demand and cultural significance contribute to its substantial economic and social impact across producing and consuming regions. The International Coffee Organization [1] reported that global coffee exports totaled 780 tons in March 2025, remaining comparable to the figures observed in March 2024. In Mexico, coffee production for the 2023–2024 marketing year was approximately 245 tons, ranking the country as the 10th largest coffee producer globally with over 2% of global coffee output [2]. Mexico’s coffee cultivation spans 14 states, with most of the production concentrated in Chiapas, Veracruz, Puebla, and Oaxaca.

In Mexico, coffee production is concentrated in four main regions: the Gulf of Mexico, Soconusco, the Pacific slope, and the north–central zone, each characterized by distinctive edaphoclimatic conditions that influence the phytochemical composition of the beans. In the north–central zone, Chiapas alone accounts for more than 40% of the national yield, underscoring its importance as a benchmark for Mexican coffee. Coffee here is typically cultivated at altitudes of 1200–1700 m along the Sierra Madre de Chiapas, where volcanic soils and cool, humid conditions favor beans with high aromatic complexity and acidity. On the Pacific slope, Colima exemplifies the coastal-to-mountain gradient, with plantations ranging from 800 to 1200 m, where sub-humid tropical weather and fertile soils generate beans with balanced body and sweetness. Oaxaca, another key Pacific producer, benefits from mountainous terrains at 1000–1600 m and temperate, humid climates, conditions that promote high-quality beans. Additionally, the highlands of Hidalgo, located in the Sierra Madre Oriental, constitute a smaller but distinctive production zone, ranking sixth nationally and contributing approximately 10% of total output. In this region, coffee is grown between 1000 and 1400 m under temperate conditions with marked seasonality, which influences both bean density and polyphenolic content [3,4,5]. In this regard, by comparing regions that dominate national yield with those that are ecologically distinct, a comprehensive perspective can be obtained on how geographical, climatic, and cultural variability shape the bioactive composition of Mexican coffee.

Additionally, with respect to geographical location and regional climate, other factors influence the quality of coffee, such as grain maturation, cultivation practices, and harvesting methods; the bean’s genetic characteristics; and the processing (i.e., roasting and grinding) techniques [6,7,8]. Roasting is a key determinant of coffee’s sensory profile, as Maillard reactions and caramelization generate a complex mixture of volatile and non-volatile compounds responsible for its flavor and aroma [9]. In addition, the degree of roasting significantly modifies coffee’s chemical composition, which in turn influences the biological effects of the brew [8,10,11,12]. Among the main bioactive components are phenolic acids such as chlorogenic, ferulic, and caffeic acids; flavonoids including epicatechin, catechin, and anthocyanins; and alkaloids such as trigonelline and caffeine [13,14]. Notably, phenolic acids and flavonoids decline as roasting progresses, while Maillard reactions promote the formation of melanoidins, which represent new bioactive compounds with distinct physiological properties [15,16]. All these compounds have been associated with diverse physiological effects, including antioxidant, neuroprotective, lipid-lowering, and hypoglycemic activities [17,18,19,20].

Evidence indicates that the degree of roasting modulates coffee’s health-protective properties; however, the effects are not uniform and vary depending on the metabolic pathway involved, the target tissue, and the biomarkers analyzed. For example, in high-fructose and saturated fat-fed rats, intense roasting enhanced anti-inflammatory responses, whereas antioxidant activity varied by tissue and parameter [21]. Similarly, coffee consistently improved body weight control, insulin sensitivity, and hepatic steatosis, with unroasted (UN) coffee showing unique benefits in reducing adipose hypertrophy, suggesting that different compounds formed or lost during roasting may target distinct physiological pathways [22]. Comprehensive preclinical studies indicate that higher roasting intensities can reduce postprandial triglycerides (TGs) and body weight gain, suggesting a potential role for darker roasts in modulating lipid metabolism and weight management [23]. These studies collectively indicate that both roasting degree and coffee composition are key determinants of bioactivity, yet gaps remain regarding the effects of roasting on digestive enzyme inhibition and nutrient absorption and the influence of regional origin on these properties.

Building on these insights, the present study aimed to provide a more comprehensive evaluation by examining *Coffea arabica* beans from four Mexican regions across different roasting degrees. We assessed in vitro antioxidant activity and digestive enzyme inhibition, alongside in vivo effects on glucose tolerance and lipid metabolism, to elucidate how regional origin and roasting intensity modulate the biological activity of coffee.

## 2. Results

The coffee brew yields were approximately 14% for all samples, regardless of the degree of roast of coffee brew samples, i.e., UN, medium roasted (MR), and high roasted (HR). This yield was equivalent to ~9 mg of dry matter per mL of coffee brew.

Moisture decreased from 9–10% to 5–7% in the MR and 3–4% in the HR samples, with no significant differences depending on coffee origin (Table 1).

Regarding color changes, L* decreased as a function of the roast degree, reflecting the darker color assumed by coffee seeds as browning proceeded during roasting. The increase in a* values accounted for the transition from the greenish hue of raw beans to the reddish-brown tones of roasted coffee. The decrease in b* with increasing roast intensity indicates the loss of the characteristic yellow hue of UN beans, while the concurrent reduction in hue angle confirms the overall shift toward darker, red-brown shades. Taken together with the decrease in L*, these parameters reflect the formation of melanoidins and other pigments during the Maillard reaction, driving the typical color development in roasted coffee (Table 1).

Spectrophotometric data confirmed the progressive development of Maillard reaction products during roasting (Table 1). Overall, absorbance at 280 nm (early products) increased from UN to HR samples. However, in coffee samples from Oaxaca and Colima, values peaked at the medium roast stage before declining at higher intensities. This indicates the subsequent degradation or transformation of the early intermediates. The intermediate products (360 nm) decreased during roasting across all regions, reflecting their consumption as precursors of more advanced compounds. In contrast, absorbance at 420 nm, which is associated with melanoidin formation, increased markedly with roast degree in all coffees.

The phenolic profile is summarized in Table 2. The main compounds detected were phenolic acids (e.g., gallic, caffeic, chlorogenic acids) and flavanols (e.g., quercetin). Notable differences were observed in UN samples; for instance, in the UN coffee from Chiapas, the concentration of gallic acid (~64.5 µg/g) was 11%, 23%, and 40% higher than in samples from Hidalgo, Oaxaca, and Colima, respectively. Likewise, the caffeic acid concentration (~1126 µg/g) was 70%, 92%, and 90% higher compared with the same regions. In contrast, coumaric acid (~315 µg/g) was lower in samples from Hidalgo (~39%), Oaxaca (~91%), and Colima (~77%) relative to Chiapas coffee samples. The quercetin glucuronide concentration (~5128 µg/g) in coffee from Chiapas was 34% and 55% higher than in samples from Hidalgo and Colima, respectively.

On the other hand, UN samples from Hidalgo and Chiapas contained the highest levels of chlorogenic acid (~233,295 and ~231,950 µg/g, respectively), whereas samples from Colima and Oaxaca showed lower concentrations by 31% and 52%, respectively, compared with the other regions.

Regarding the UN samples, coffee from Oaxaca showed the highest levels of quercetin (~56,290 µg/g), whereas samples from Chiapas, Hidalgo, and Colima were lower by ~13%, 85%, and 99%, respectively. Finally, UN coffee from Colima exhibited higher levels of 2,4,6-trihydroxybenzoic acid, ferulic acid, and t-cinnamic acid compared with the other regions.

The profile of bioactive compounds varied with roasting degree, showing a progressive decrease from UN to HR samples. For example, in coffee from Chiapas, gallic acid decreased by 14% at MR and was completely absent at HR. Similarly, caffeic acid was reduced by 94% at MR and fully degraded at HR, while quercetin decreased by 95% at MR and 99% at HR. Despite these reductions, these compounds remained among the most abundant in MR coffee samples from Chiapas.

A similar effect was observed in the coffee samples from Hidalgo, where the predominant compound, chlorogenic acid, decreased by 83% in MR to 98% in HR samples. In Oaxaca, quercetin was almost completely degraded (99% and 100% reductions in MR and HR, respectively), while quercetin glucuronide decreased by 67% in MR to 69% in HR compared with UN samples. Finally, in the coffee from Colima, the main compounds also showed marked reductions in the MR and HR samples: 2,4,6-trihydroxybenzoic acid (38% and 100%), ferulic acid (90% and 99%), and t-cinnamic acid (11% and 100%).

**Figure 1:**
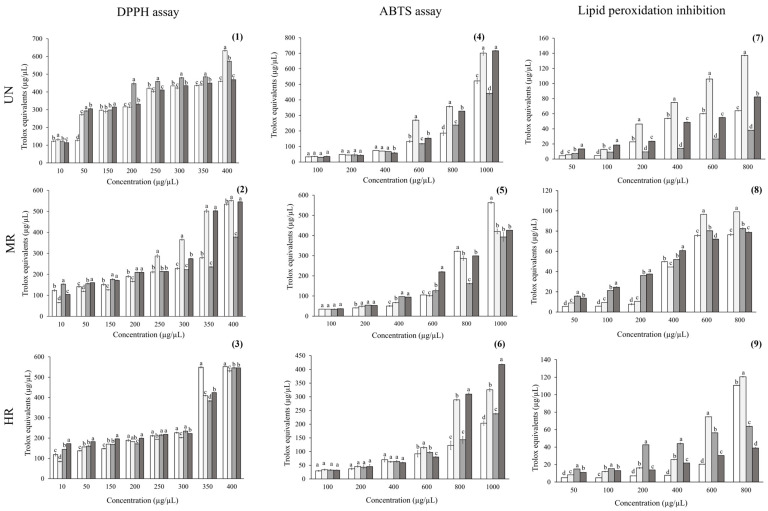
Antioxidant assessment of coffee brews from 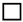 Chiapas (CH), 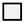 Colima (CO), 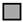 Oaxaca (O), and 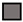 Hidalgo (H) with respect to unroasted (UN), medium-roast (MR), and high-roast (HR) brews. (**1**–**3**) DPPH radical scavenging; (**4**–**6**) ABTS assay, and (**7**–**9**) lipid peroxidation inhibition. Values are expressed as mean ± standard error. Different letters indicate statistically significant differences among coffee samples within each region at the same concentration (*p* < 0.05; ANOVA with Tukey’s post hoc test).

The results of the antioxidant activity of the coffee brews are shown in Figure 1. Antioxidant effects varied according to both the region and roasting degree. The DPPH assay revealed that, at the highest concentration, UN coffee from Colima exhibited the greatest scavenging capacity, with values significantly higher than those from Chiapas (*p* = 0.02), Hidalgo (*p* < 0.001), and Oaxaca (*p* < 0.001). For MR, coffee from Colima maintained the highest effect, followed closely by coffee from Hidalgo, with no significant differences between them (*p* > 0.05). In contrast, coffee from Chiapas and Oaxaca showed lower activity. Finally, at the HR degree, the antioxidant potential of Colima coffee decreased compared with the other samples, while coffee from Chiapas displayed significantly greater activity than coffee from both Oaxaca (*p* < 0.001) and Hidalgo (*p* < 0.001). These findings confirm that both the roasting degree and geographic origin play a decisive role in the antioxidant capacity of Mexican coffee samples.

In the ABTS assay, UN coffee from Colima and Hidalgo exhibited the highest activities at 1000 µg/µL (~700 and ~650 µg Trolox equivalents/µL, respectively), whereas samples from Chiapas and Oaxaca showed the lowest values (~480–400 µg Trolox equivalents/µL). At the medium-roast degree, coffee from Chiapas stood out with significantly higher activity (~580 µg Trolox equivalents/µL) compared with samples from the other regions (420–500 µg Trolox equivalents/µL). At the high-roast degree, coffee from Hidalgo (~420 µg Trolox equivalents/µL) and Colima (~400 µg Trolox equivalents/µL) retained the greatest activities, while Chiapas (~300 µg Trolox equivalents/µL) and Oaxaca (~280 µg Trolox equivalents/µL) exhibited a marked loss of activity.

In lipid peroxidation inhibition, UN coffee from Colima consistently exhibited the greatest activity (~140 µg Trolox equivalents/µL at 800 µg/µL), followed by Hidalgo (~110 µg Trolox equivalents/µL), while Chiapas (~100 µg Trolox equivalents/µL) and Oaxaca (~40 µg Trolox equivalents/µL) showed the lowest activities. At the MR degree, Colima coffee maintained the highest effect, followed by Oaxaca, whereas Chiapas and Hidalgo were statistically similar (*p* < 0.001). At the high roast degree, Colima retained its superior activity (~130 µg Trolox equivalents/µL), followed by Chiapas, which significantly increased at 1000 µg/µL (~120 µg Trolox equivalents/µL). In contrast, Oaxaca and Hidalgo exhibited the lowest values (~70–35 µg Trolox equivalents/µL).

**Figure 2:**
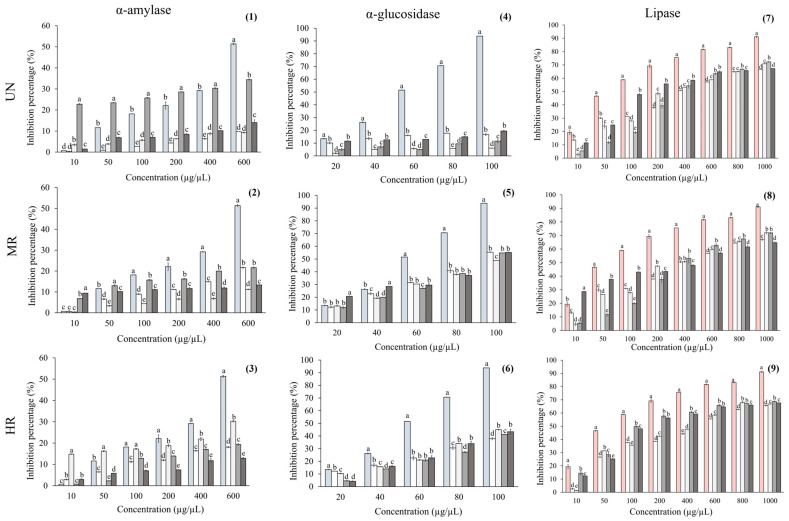
Digestive enzyme assessment of coffee brews from 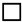 Chiapas (CH), 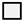 Colima (CO), 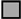 Oaxaca (O), and 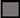 Hidalgo (H) of unroasted (UN), medium-roast (MR), and high-roast (HR) brews. 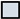 Acarbose or 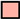 orlistat was used as a positive control. (**1**–**3**) Lipase; (**4**–**6**) α-amylase, and (**7**–**9**) α-glucosidase inhibition assessment. Data are presented as mean ± standard error. Different letters indicate statistically significant differences among coffee samples within each region at the same concentration (ANOVA with Tukey’s test, *p* < 0.05).

The results for digestive enzymes are shown in Figure 2. For lipase activity inhibition, the positive control (orlistat) exhibited 91% of enzyme inhibition at the highest concentration tested, whereas all coffee samples showed lower inhibition percentages (~67–70%). As a result, all coffee samples exhibited ~5-fold higher median inhibitory concentration (IC_50_) values compared to orlistat (Table 3).

For α-amylase inhibition, all coffee brews exhibited lower percentages than the positive control (acarbose). Among UN samples, coffee from Oaxaca showed the highest inhibition (34%, *p* < 0.001), although this activity decreased markedly at MR and HR degrees (~0.6-fold). UN coffees from Chiapas and Colima displayed similar inhibition (~10%, *p* > 0.05). In coffee from Chiapas, inhibition increased at MR (~2-fold), whereas coffee from Colima remained unchanged, and with respect to HR, an increase of ~3-fold was observed. In contrast, coffee from Hidalgo consistently exhibited the lowest inhibition percentages across all roast degrees.

Regarding IC_50_ results, all coffee samples exhibited higher values than the positive control, ranging from ~9- to 124-fold. Among the coffee types, UN samples from Chiapas showed ~2.2-fold lower IC_50_ values compared with MR and HR samples. In Colima, MR samples displayed ~2.7-fold lower IC_50_ values than UN and HR samples. For Oaxaca, HR samples had lower IC_50_ values than both UN (~13-fold) and MR samples (~7.7-fold). Finally, in Hidalgo, MR samples showed lower IC_50_ values than UN (~2.6-fold) and HR samples (~3.8-fold) (Table 3).

All coffee brews exhibited lower α-glucosidase inhibition than acarbose, which reached 93% inhibition at the highest concentration (Figure 2). Among the UN samples, inhibition percentages ranged from ~6% to 19%. These values increased substantially at the MR degree, reaching ~55% across all samples, but decreased again at the HR degree to ~27%.

Regarding IC_50_ results, all samples showed higher values than the positive control (acarbose), where all UN coffee samples showed the highest values, ranging from ~56 to 340-fold. On the other hand, for all roasted coffee, the IC_50_ values decrease from ~2-fold compared with the control (Table 3).

**Figure 3:**
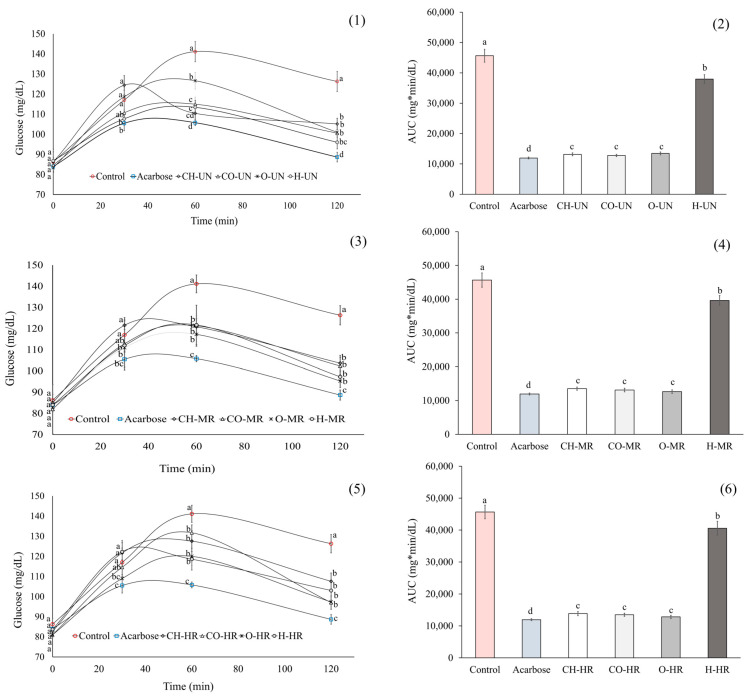
Oral Starch Tolerance Test (OSTT) and area under the curve (AUC) of coffee brews from Chiapas (CH), Colima (CO), Hidalgo (H), and Oaxaca (O): (**1**,**2**) unroasted (UN), (**3**,**4**) medium-roast (MR), and (**5**,**6**) high-roast (HR) brews, respectively. Data are presented as mean ± standard error. Distinct letters denote significant group differences (ANOVA with Tukey’s test, *p* < 0.05).

Figure 3 presents the Oral Starch Tolerance Test (OSTT) results. After 120 min of starch administration, serum glucose levels were reduced in rats treated with UN, MR, or HR coffee by approximately 20%, 21%, and 18%, respectively, compared with the negative control group. However, such a decrease was smaller than that observed with the positive control (acarbose) (~13% for UN, ~12% for MR, and ~14% for HR). Even so, these results were consistent with the area-under-the-curve (AUC) values, with coffee samples from Hidalgo showing the highest AUC values (Figure 3).

**Figure 4:**
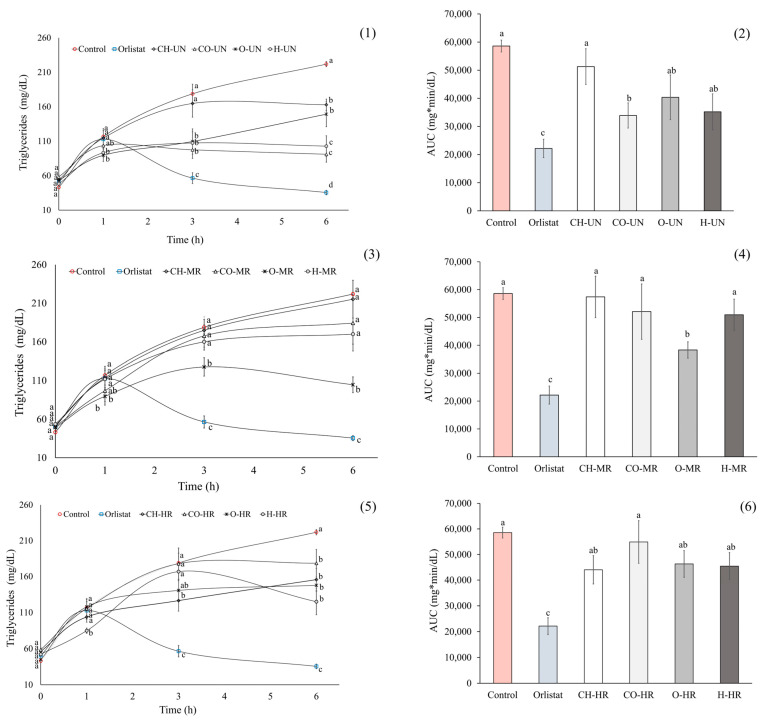
Oral Lipid Tolerance Test (OSTT) and area under the curve (AUC) of coffee brews from Chiapas (CH), Colima (CO), Hidalgo (H), and Oaxaca (O): (**1**,**2**) unroasted (UN), (**3**,**4**) medium-roast (MR), and (**5**,**6**) high-roast (HR) brews, respectively. Data are presented as mean ± standard error. Distinct letters denote significant group differences (ANOVA with Tukey’s test, *p* < 0.05).

With respect to roasted coffee samples, although a decrease in serum TG levels was observed after lipid load, the effect was smaller than the UN coffee samples. Nevertheless, serum TG levels remained lower than the negative control, particularly in the HR samples, with the strongest effects observed for Oaxaca (~49% for MR and ~31% for HR) and Hidalgo (~21% for MR and ~41% for HR). Regarding the AUC values, only the MR coffee from Oaxaca exhibited a reduction compared with the negative control (Figure 4).

Regarding the Oral Lipid Tolerance Test (OLTT), UN coffee from all regions reduced serum TG levels relative to the control group by ~26% (Chiapas), ~58% (Colima), ~32% (Oaxaca), and ~54% (Hidalgo). However, these effects were smaller than those of orlistat (~4.5-fold for Chiapas, ~2.5-fold for Colima, ~4.2-fold for Oaxaca, and ~2.8-fold for Hidalgo) (Figure 4). For the AUC values, only coffee samples from Colima showed a reduction compared with the negative control (Figure 4).

Finally, an analysis of roast-dependent changes on active phenolic compounds (chlorogenic acid and quercetin) and their acute bioactivity with respect to triglyceride and glucose levels according to coffee origin was performed (Supplementary Tables S1 and S2).

## 3. Discussion

Coffee has long been associated with beneficial health effects; nevertheless, the extent to which bean origin and roasting degree determine its bioactive properties remains a subject of debate. In this regard, we examined whether variations in roasting intensity and coffee origin from selected Mexican regions affect bioactive potential. Accordingly, coffee samples from Chiapas, Colima, Oaxaca, and Hidalgo were selected for this study, and their brews were prepared following methods previously described for Mexican coffee [24].

Our results demonstrated that moisture content and color changes upon roasting were consistent with the literature’s values, with decreases in L*, hue angle, and moisture reflecting the typical evolution occurring upon roasting, which is well known in the literature [9,25]. Indeed, differences among origins highlight the influence of bean composition on the extent of these changes that may affect the composition of bioactive compounds and their activity.

Coffee brews are well established as a rich source of bioactive compounds, especially polyphenols such as phenolic acids, with chlorogenic, ferulic, and p-coumaric acids being the most representative. In addition, previous studies have shown that both the production system and roasting degree (light, medium, or dark) significantly affect the polyphenolic profile of coffee [26]. In agreement with these findings, our results show that UN coffees were especially rich in phenolic acids and flavonoids, with clear regional differences. Specifically, samples from Chiapas contained the highest levels of caffeic and gallic acids, whereas samples from Hidalgo contained the highest chlorogenic acid, and those from Oaxaca and Colima had the highest quercetin and ferulic and cinnamic derivatives, respectively. These differences reflect the impact of edaphoclimatic factors on the phytochemical profile of Mexican coffees, consistent with previous reports describing regional effects on phenolic accumulation [27]. Beyond compositional differences, the antioxidant assays mirrored the regional profiles, highlighting the functional relevance of specific compounds. Coffee samples from Colima consistently exhibited the strongest inhibition of lipid peroxidation, likely related to its higher ferulic and cinnamic acid content, for which its radical-scavenging and membrane-protective activities are well documented [28]. Coffee from Chiapas retained the highest activity in the DPPH assay at HR, suggesting that despite gallic and caffeic acid degradation, its residual phenolic pool and/or melanoidin formation sustained antioxidant potential [29,30]. In contrast, the coffee samples from Hidalgo, which are rich in chlorogenic acids, showed the steepest decline after roasting, consistent with their thermal instability [31,32]. Coffee from Oaxaca maintained competitive ABTS activities in UN and HR conditions, probably due to its initial quercetin abundance and the contribution of Maillard-derived antioxidants [33,34]. These findings suggest that coffee’s antioxidant potential depends not exclusively on the absolute levels of native phenolic compounds but rather on the dynamic equilibrium between their thermal degradation and the concomitant formation of secondary products during roasting. Melanoidins and other Maillard reaction products, despite exhibiting lower radical-scavenging capacity than chlorogenic acids, contribute significantly through pathways involving metal chelation and reactive radical quenching, thereby sustaining measurable antioxidant activity even in high-roast samples [35,36]. The fact that coffee samples from Chiapas and Colima preserved higher antioxidant activity in roasted brews suggests that their chemical profiles favored the retention or transformation into more stable antioxidant structures. In this regard, our results align with the changes in MRI, which revealed that roasting promotes the accumulation of early Maillard products and the subsequent generation of advanced melanoidins, both recognized for their antioxidant activity [14,37]. For instance, MRI absorbance at 420 nm, associated with melanoidin formation, increased ~4.2-fold in Chiapas coffee at MR and ~9-fold at HR, whereas in Colima coffee, the increase was ~4.2-fold for both MR and HR. In contrast, the overall decrease in intermediate compounds across all origins reflects their consumption and emphasizes their role in generating more complex structures. Overall, the MRI data suggest that regional differences in the composition of UN coffee influence the balance between the early, intermediate, and advanced Maillard pathways, thereby modulating the antioxidant profile of coffee brews.

Coffee’s anti-hyperglycemic and anti-hyperlipidemic properties are thought to be mediated, at least in part, through the inhibition of key digestive enzymes involved in carbohydrate metabolism (e.g., α-amylase and α-glucosidase) and lipid metabolism (e.g., lipase) [16,19]. Moreover, previous studies have shown that coffee’s bioactive properties are influenced not only by roasting degree but also by the region of cultivation [15]. To our knowledge, this is the first study to compare coffees from four Mexican regions with respect to their effects on digestive enzymes. Our results demonstrated that the most significant differences in enzyme inhibition occurred primarily between regions, followed by variations due to roasting. For example, in lipase inhibition assays, all samples exhibited inhibition percentages ranging from 67 to 70%, with slight increases observed in some cases after roasting, particularly at the MR. In amylase assays, the effect of roasting varied by region: Inhibition decreased in Colima samples but increased in Oaxaca samples at HR degrees. For glucosidase inhibition, all samples showed an increase in activity with HR. Our results agree with previous studies on coffees from other regions, specifically Vietnam, which have reported that the inhibition of digestive enzymes, such as glucosidase, correlates with the roasting degree, consistent with the complexity of coffee composition [16]. In this regard, we also attribute the biological effect observed to differences in the composition of bioactive compounds among regions, as well as to the synthesis of novel molecules during the roasting process.

Considering that chlorogenic acids are the most abundant phenolic compounds in UN coffee—accounting for nearly ~80% of its total phenolic content—and are regarded as the main contributors to its bioactivity [38,39], roasting promotes their degradation into derivatives such as 3-caffeoylquinic acid and 5-caffeoylquinic acid, leading to reductions ≥ 90% of the initial chlorogenic acid content depending on roasting intensity and thereby diminishing overall bioactivity [16]. Accordingly, we detected a marked decline in these compounds across all analyzed samples. In contrast, the roasting process favors the generation of Maillard reaction products, which are known to influence coffee bioactivity [16,40,41]. In agreement with our observations, previous research has documented enhanced α-glucosidase inhibition with increasing roasting intensity, an effect that appears to stem from the formation of Maillard-derived compounds [19,21].

Our in vivo results on carbohydrate absorption showed that all groups receiving coffee brews exhibited a ~20% reduction in glucose levels, regardless of crop region or roasting degree. Similar findings were previously reported for Veracruz coffee samples with different roasting intensities [23]. Although the reduction in postprandial glucose induced by coffee brews was lower than that achieved with the positive control, comparable decreases in postprandial glucose have been observed with other natural products, supporting their potential as complementary strategies for managing hyperglycemia in diabetes [42].

To explore whether these effects were related to the main identified compounds in Mexican coffee, we analyzed roast-dependent changes in chlorogenic acid and quercetin, as well as their acute bioactivity, according to coffee origin; no significant associations were found. As it is difficult to attribute biological effects to a single compound, our results strongly suggest that other constituents, such as Maillard reaction products and additional polyphenols, which were not evaluated in this study, may contribute to the observed activity. Therefore, further studies are needed to clarify the mechanisms underlying these effects.

With respect to the lipid-lowering activity of coffee, our findings indicate that both roasting degree and crop region modulate bioactivity, with the strongest effects observed in HR samples from Hidalgo and Oaxaca, although these were still less effective than orlistat. Interestingly, and in contrast to the carbohydrate absorption results, we found significant associations between roast-dependent changes in chlorogenic acid and quercetin and the acute bioactivity by coffee origin, suggesting that these compounds may partly contribute to the observed lipid-lowering effects.

The reduction in lipid absorption observed in this study may be partially ascribed to the lipase-inhibitory activity evidenced in the in vitro assays. In this regard, previous investigations have demonstrated that bioactive constituents of coffee attenuate dietary lipid digestion through multiple mechanisms. Among these, inhibition of digestive lipase has been recognized as a principal pathway, as reported in both in vitro and in vivo models [17,43,44]. For example, trigonelline inhibited digestive enzymes that included pancreatic lipase in rats, while in in vitro reports, other compounds such as caffeoylquinic and feruloylquinic acids and their isomers were found to reduce the interfacial area of lipid emulsions and promote an increase in droplet size [45]. Additionally, coffee bioactive compounds can interfere with bile acid activity, which is essential for lipid emulsification and digestion. In vitro studies have demonstrated that CGAs interact with bile acids, thereby impairing their emulsifying capacity [17]. Likewise, cafestol has been shown to suppress bile acid synthesis in rodents, which may in turn affect lipid digestion [46,47]. Collectively, these findings support that the inhibition of both lipase activity and bile acid function by coffee bioactive compounds may contribute to reduced lipid digestion. Nonetheless, further studies are needed to fully elucidate the underlying mechanisms of lipid digestion inhibition as potential anti-obesity targets.

### Limitations of This Study

The chemical characterization in this study focused on identifying specific compounds for which authentic standards were available (phenolic acids and selected flavonoids), allowing us to confirm their presence and quantify them accurately. We acknowledge that additional unidentified peaks were observed in the chromatograms, which are of great interest for future investigation. Subsequent studies could include a more comprehensive analysis using a broader set of reference standards and advanced identification strategies (e.g., tandem MS/MS, molecular networking, databases) to expand the chemical profile of the samples. Furthermore, incorporating other bioactive compounds such as trigonelline, caffeoylquinic acids, feruloylquinic acids, and cafestol would provide a broader phytochemical profile and help clarify potential mechanisms of action, including lipid digestion inhibition.

## 4. Materials and Methods

### 4.1. Coffee Roasting Process and Physical Characterization

The UN coffee beans (*Coffea arabica* L.) from the 2020 harvest in Chiapas, Oaxaca, Hidalgo, and Colima, México, were generously donated by local farmers. Samples were identified in the herbarium “Jorge Arturo Alba Avila de la Facultad de Ciencias Biológicas de la Universidad Juárez del Estado de Durango” and assigned voucher number HJAAA/FCB-UJED/06-2025-0002 in the collection of cultivated plants. The green coffee beans were processed according to the traditional post-harvest practices of the local farmers before roasting, which included dry natural process under the sun; the cherries were pulped; the mucilage was partially removed before drying.

Coffee roasting conditions often vary by region or country. In this study, we evaluated roasting parameters based on the traditional practices of the local farmers who donated the samples, thereby reflecting the conditions under which the coffee is typically commercialized. In this regard, roasting was performed in an oven (MT Maquinaria, Puebla, Mexico) equipped with a needle valve for gas pressure regulation, enabling accurate temperature control, and an air-based cooling system to ensure stable roasting conditions. Medium roasting (MR) was achieved by maintaining hot air circulation at 240 °C for 4 min 10 s, whereas high roasting (HR) was obtained by extending the roasting duration to 5 min 10 s. Following roasting, samples were rapidly cooled and subsequently ground using a mill (Hamilton Beach 80335Rv, Richmond, VA, USA) fitted with a sieve to a standard particle size of ~0.850 mm, corresponding to that commonly employed for commercial coffee.

Coffee brews were prepared following the methodology described by Gallardo-Ignacio et al. [24]. In brief, extraction was carried out using a French press with 6.6 g of coffee powder (approximately one tablespoon) per 100 mL of water. The resulting brews were freeze-dried using a FreeZone 18 Liter Console Freeze Dry System (Labconco Corporation, KS, USA), and the lyophilized samples were stored in amber containers until further use.

#### 4.1.1. Yield

The coffee brew yield was determined as follows:

Yield _(%)_ = [(lyophilized coffee brew_(g)_)/(coffee powder_(g)_] *×* 100 (1)

The results are reported as means of two independent preparations.

#### 4.1.2. Moisture

Coffee moisture was determined by the gravimetric method [48]. Briefly, ground coffee (1 g) samples were weighed before and after overnight drying at 75 °C under vacuum (Vuotomatic 50, Bicasa, Milano, Italy), and moisture was expressed as the percentage ratio between weight loss and the initial weight.

#### 4.1.3. Coffee Color

The color of ground coffee was determined using a tristimulus colorimeter (Chromameter-2 Reflectance, Minolta, Osaka, Japan) equipped with a CR-300 measuring head, which was standardized against a white calibration tile prior to measurement. Color values were expressed in CIE coordinates: L (lightness/darkness), a (redness/greenness), and b* (yellowness/blueness). The a* and b* parameters were subsequently used to calculate the hue angle (tan^−1^ b*/a*).

#### 4.1.4. Spectrophotometric Analysis

Non-specific markers of the Maillard reaction were assessed by measuring absorbance at 280 nm (early non-colored compounds), 360 nm (intermediate compounds), and 420 nm (high-molecular-weight products, primarily melanoidins) using a UV–VIS Recording Spectrophotometer (UV-2501PC, Shimadzu Corporation, Kyoto, Japan) in appropriately diluted coffee brews. Maillard reaction indices (MRIs) at each wavelength were calculated by multiplying the absorbance values by the corresponding dilution factor [41].

#### 4.1.5. Phenolic Profile by Ultra Performance Liquid Chromatography (UPLC)

For the identification and quantification of the phenolic profile of coffee samples, we followed the method described by Díaz-Rivas et al. [49]. Data were collected in the multiple reaction monitoring (MRM) mode. Data acquisition and processing were performed using MassLynx version 4.1 software (Waters Corp., Milford, MA, USA).

Chromatographic separations were carried out using a C18 Acquity UPLC BEH column (100 mm × 2.1 mm × 1.7 µm) (Waters Corp., Milford, MA, USA) operating at 35 °C, with 7.5 mM water/formic acid (A) and acetonitrile (B) as mobile phases at a flow rate of 0.35 mL/min and a sample injection volume of 2 µL. The gradient was applied as follows: 3% B, held for 1.23 min; 9% B at 3.82 min; 16% B at 11.40 min; 50% B at 13.24 min; and 3% B at 15 min, returning to the initial conditions (3% B). Negative ionization was used for MS, with ESI conditions as follows: capillary voltage, 2.5 kV; desolvation temperature, 300 °C; source temperature, 150 °C; desolvation and cone gas flow rates, 500 L/h and 151 L/h, respectively; and collision gas flow rate, 0.13 mL/min. MRM transitions were determined via MS/MS spectra for both the standards and the polyphenols present in the samples. Peak identification was based on the comparison of retention times and MRM transitions with those of pure standards. Quantitative determinations of phenolic compounds were performed using calibration curves of standards obtained from Sigma Chemical (St. Louis, MO, USA).

### 4.2. Antioxidant Capacity

Antioxidant activity was evaluated using the DPPH and ABTS assays.

#### 4.2.1. DPPH Assay

DPPH was performed following the methodology of Xu and Chang [50]. Briefly, 0.2 mL of coffee samples at concentrations ranging from 10 to 400 µg/mL was added to 3.8 mL of an ethanol solution of DPPH radicals (final concentration: 0.1 mM). The mixture was vortexed for 1 min and then incubated at room temperature in the dark for 30 min. The ability of the coffee samples to inhibit the DPPH radical was determined by a decrease in absorbance at 517 nm using a spectrophotometer (Spectronic^®^ 20 Genesys™, Spectronic Instruments, Thermo Scientific, Waltham, MA, USA). The results were expressed as Trolox equivalents, calculated from a calibration curve prepared with Trolox under the same conditions as the samples (Sigma Chemical Co., St. Louis, MO, USA).

#### 4.2.2. ABTS Assay

The ABTS assay was conducted according to Re et al. [51]. The ABTS [2,2′-azino-bis (3-ethylbenzothiazoline-6-sulfonic acid)] was dissolved in water to a final concentration of 7 mM. The ABTS radical cation (ABTS•^+^) was generated by reacting the ABTS stock solution with 2.45 mM potassium persulfate and allowing the mixture to stand in the dark at room temperature for 12–16 h before use. The ABTS•^+^ solution was then diluted with PBS (pH 7.4) to an absorbance of 0.70 at 734 nm and equilibrated at 30 °C. Coffee samples (1.0 mL) at concentrations ranging from 100 to 1000 µg/µL were mixed with 1.0 mL of the ABTS•^+^ solution. Absorbance was recorded at 734 nm, at 30 °C, exactly 1 min after initial mixing and up to 6 min. The results were expressed as Trolox equivalents, calculated from a calibration curve prepared with Trolox under the same conditions as the samples.

#### 4.2.3. Lipid Peroxidation

Lipid peroxidation was assessed using the procedure described by Rocha-Guzmán et al. [52]. Briefly, 600 μL of serum from healthy rats was mixed with 300 μL of coffee samples at concentrations ranging from 50 to 800 µg/mL in a phosphate buffer (pH 7.4). Subsequently, 150 μL of 500 mM H_2_O_2_ and 150 μL of 100 mM FeCl_3_ were added to induce Fenton’s reaction, and the mixture was incubated for 3 h at 37 °C. After incubation, 1 mL of each sample was mixed with 4.0 mL of TBA reagent (40.5 mL of 20% acetic acid adjusted to pH 3.5 with 1 M NaOH, 13.2 mL of 8.2% SDS, and 40.5 mL of 0.8% TBA and then brought to 100 mL with double-distilled water) and heated at 96 °C for 80 min. The samples were then cooled on ice, mixed with 5 mL of n-butanol, and centrifuged at 3000× *g* for 15 min. The ability of the samples to inhibit plasma oxidation was determined by the decrease in absorbance at 532 nm using a spectrophotometer (Spectronic^®^ 20 Genesys™, Spectronic Instruments, Thermo Scientific, MA, USA). The results were expressed as Trolox equivalents, calculated from a calibration curve prepared with Trolox under the same conditions as the samples.

### 4.3. Digestive Enzyme Inhibition Assessment

#### 4.3.1. Pancreatic Lipase

The assay was conducted according to the methodology of McDougall et al. [53], with minor modifications. In brief, 150 μL of a 10 mg/mL solution of porcine pancreatic lipase type II (SIGMA Co., St. Louis, MO, USA) was combined with coffee samples at concentrations ranging from 10 to 1000 µg/µL, together with 400 μL of Tris buffer (100 mM, pH 8.2), 400 μL of p-nitrophenyl laurate (SIGMA Co., St. Louis, MO, USA), and the substrate solution (0.08%, *w*/*v*, prepared in 5 mM sodium acetate, pH 5.0, containing 1% Triton X-100). The mixtures were incubated at 37 °C for 2 h and subsequently centrifuged at 16,000 rpm for 3 min. Absorbance of the supernatant was recorded at 400 nm using a microplate reader (MultiScan Go, Thermo Scientific, MA, USA). A dose–response curve was constructed using orlistat (Redustat^®^; Laboratorios Liomont S.A. de C.V., CDMX, Mexico) as the reference inhibitor, and the results were expressed as equivalents of the positive control. The minimum inhibitory concentration (IC_50_) value for each coffee extract was determined by plotting the percentage of inhibition against the log-transformed concentrations of the samples [54].

The inhibition percentage was calculated with the following equation:

Inhibition _(%)_ = [λ_0_ − λ_1_)/λ_0_] *×* 100 (2)

where λ_0_ is the absorbance of the blank, and λ_1_ is the absorbance of each coffee sample.

#### 4.3.2. α-Amylase

α-Amylase inhibition was evaluated following the method of Tamil et al. [55]. Coffee samples (10–600 µg/µL) were incubated with 2.5% gelatinized starch and 100 μL of porcine pancreas α-amylase solution (2.5 mU/μL in sodium phosphate buffer 0.02 M and NaCl 6 mM at pH 6.9) (SIGMA Co., St. Louis, MO, USA) at 37 °C for 1 h, and the glucose concentration in the supernatant was determined using an enzymatic kit (Biosystems, Barcelona, Spain). Acarbose was used as the positive control, and IC_50_ values were determined by plotting inhibition percentages against log-transformed sample concentrations.

#### 4.3.3. α-Glycosidase

The assay was performed according to the method described by Apostolidis et al. [56]. Briefly, 25 μL of each coffee sample (20–100 µg/µL) was combined with 100 μL of α-glucosidase solution (0.19 mU/µL; SIGMA Co., St. Louis, MO, USA) and 50 μL of phosphate buffer (0.1 M, pH 6.9), followed by incubation at 37 °C for 10 min. Subsequently, 25 μL of the p-nitrophenyl-α-D-glucopyranoside solution (5 mM, prepared in 0.1 M citrate–phosphate buffer, pH 7; SIGMA Co., St. Louis, MO, USA) was added, and the mixture was incubated for an additional 30 min at 37 °C. The reaction was terminated by adding 1 mL of 0.05 M NaOH, and absorbance was recorded at 410 nm using a Synergy HT Microplate Reader (Biotek Instruments, Inc., Wiesbaden Germany). A dose–response curve was generated using acarbose (Laboratorios Alpharma, S.A. de C.V., CDMX, Mexico) as the reference inhibitor, and the results were expressed relative to this positive control. IC_50_ values were calculated by plotting the percentage of inhibition against the log-transformed concentrations of coffee samples.

### 4.4. Carbohydrates and Lipids Absorption Inhibition Assessment

#### 4.4.1. Animal Subjects and Ethical Approval

All experimental procedures were carried out in compliance with the guidelines of the National Institutes of Health [57] and the Mexican Official Norm [58]. The protocol was reviewed and approved by the Research Committee of the Mexican Social Security Institute (R-2023-785-067). Female Wistar rats (N = 40; 200 ± 20 g) were used in this study. The sample size was determined considering a minimum of individuals for similar animal experiments [23].

Animals were housed under controlled environmental conditions with regulated light and temperature, following a 12 h light/dark cycle at 25 ± 1 °C. A one-week acclimatization period was provided prior to the start of the experiment. During both the acclimatization and experimental phases, rats had ad libitum access to rodent chow 5001 (Purina^®^, Québec, QC, Canada). The experimental unit was one animal.

Inclusion and exclusion criteria, as well as rules for data point exclusion, followed the Mexican Official Norm (NOM-062-ZOO-1999) [58]. Animals showing signs of pain or illness would have been excluded, and euthanasia would have been performed if recovery was not possible. However, no such cases occurred, and all animals completed the experiment.

Randomization was based on the initial body weight to ensure homogeneous groups at baseline. To minimize potential confounders—such as treatment order, measurement sequence, or animal and cage location—each animal was individually identified with ear tags, and cages were appropriately labeled for consistent tracking throughout the study.

The first author (C.I.G.G.) was aware of the group allocation at all stages of the study, including allocation, experimental procedures, outcome assessment, and data analysis. All outcome measures assessed were biochemical parameters, with the primary outcome being their change relative to the control groups.

#### 4.4.2. Experimental Design

The effects of coffee samples on carbohydrate and lipid digestion and absorption were assessed using the OSTT and OLTT. Ten curves were performed in total, five for each test. Two curves served as negative controls: 3 g of starch/kg body weight for the OSTT and a lipid emulsion prepared from corn oil and lard (1:1) at a dose of 10 mL/kg body weight for the OLTT. Another two curves served as positive controls: 5 mg of acarbose/kg body weight (equivalent to the typical human dose of 50 mg ingested with a meal) for the OSTT and 6 mg of orlistat/kg body weight (equivalent to the human therapeutic dose of 60 mg per meal) for the OLTT. The remaining three curves corresponded to the interventions (UN, MR, and HR coffee samples). Each curve included eight animals. Because these were acute tests, the same animals were used for both the OSTT and OLTT, with a one-week washout period between them to allow recovery and a return to baseline conditions.

The dose of coffee administered to rats was calculated based on the human equivalent intake of 240 mL of coffee brew (approximately 1.6 cups), corresponding to 2.5 g of freeze-dried coffee and considering an average adult body weight of 60 kg [24]. The conversion to the equivalent animal dose (mg/kg body weight) was performed using the following equation [59]:

R1 = (A)(KMA/KMR)(3)

where (R1) represents the animal dose equivalent, and (A) represents the adult human dose of lyophilized coffee brew (41.6 mg/kg body weight). KMA represents the conversion factor for adult humans, which is 37, and KMR represents the conversion factor for rats, which is 6. The equivalent animal dose was calculated to be 257 mg of lyophilized coffee brew per kg of body weight.

Following an overnight fast (~8 h), baseline blood samples were collected from the lateral tail vein of rats (n = 8), corresponding to time zero of the curve. Subsequently, animals received either starch for the OSTT or a lipid load for the OLTT, administered together with coffee samples, acarbose, or orlistat according to the experimental group via intragastric gavage. Post-administration, blood samples were collected at 30, 60, and 120 min for the OSTT or at 1, 3, and 6 h for the OLTT. Serum glucose and TG concentrations were determined using a commercial enzymatic kit (Biosystem Laboratories, Barcelona, Spain) according to the manufacturer’s instructions. The onset time, peak values, and AUC were analyzed to assess the relative rate of carbohydrate and lipid digestion and absorption.

### 4.5. Statistical Analysis

Data are presented as mean ± standard error (SE). Differences among study groups were assessed using one-way analysis of variance (ANOVA) followed by Tukey’s post hoc test. A *p*-value < 0.05 was considered statistically significant. All analyses were performed using SPSS version 20.0 (SPSS Inc., Chicago, IL, USA).

## 5. Conclusions

The phytochemical profile and biofunctional potential of Mexican coffees are strongly influenced by both crop region and roasting degree. Although UN coffee contained the highest concentrations of bioactive compounds, notable regional differences were observed. With roasting, this profile shifted, showing a progressive decrease from UN to HR samples. However, the biological effects assessed in this work did not depend solely on the reported bioactive compounds but were also likely influenced by compounds formed during roasting. Notably, both MR and HR, in different ways, enhanced inhibitory activity against digestive enzymes and contributed to lipid-lowering properties in vivo, and this is comparable to their UN counterparts. These findings highlight that the selection of coffee origin and roasting degree can enhance specific health-promoting properties, supporting the concept of coffee as a functional beverage with the potential to modulate oxidative stress and regulate carbohydrate and lipid metabolism. Future studies should expand phytochemical characterization beyond phenolic compounds, explore mechanisms underlying enzyme inhibition, and evaluate the long-term metabolic effects of region-specific coffees in vivo.

## Figures and Tables

**Table 1:** Moisture content, color parameters, and Maillard reaction indices (MRIs) in coffee from four Mexican regions.

	UN	MR	HR
	Coffee from Chiapas		
Moisture (%)	10.0 ± 0.03 ^a^	4.5 ± 0.01 ^b^	3.9 ± 0.00 ^c^
a*	2.1 ± 0.02 ^c^	9.0 ± 0.07 ^a^	7.2 ± 0.11 ^b^
b*	16.9 ± 0.12 ^a^	2.9 ± 0.17 ^b^	1.1 ± 0.13 ^c^
L*	65.4 ± 0.48 ^a^	27.4 ± 0.12 ^b^	23.2 ± 0.24 ^b^
Hue angle	1.4 ± 0.01 ^a^	0.3 ± 0.01 ^b^	0.1 ± 0.02 ^b^
MRI_280_	68.0 ± 1.2 ^c^	177.0 ± 7 ^b^	206.0 ± 7.5 ^a^
MRI_360_	58.0 ± 1.2 ^a^	61.0 ± 1.8 ^a^	48.0 ± 1.7 ^b^
MRI_420_	2.0 ± 0.1 ^c^	8.5 ± 0.1 ^b^	18.0 ± 0.7 ^a^
	Coffee from Colima		
Moisture (%)	8.8 ± 0.03 ^a^	4.9 ± 0.07 ^b^	3.6 ± 0.0 ^c^
a*	1.7 ± 0.03 ^b^	6.0 ± 0.06 ^a^	1.9 ± 0.11 ^b^
b*	12.6 ± 0.06 ^a^	3.4 ± 0.09 ^c^	5.3 ± 0.04 ^b^
L*	53.6 ± 0.09 ^a^	22.1 ± 0.05 ^b^	21.0 ± 0.20 ^b^
Hue angle	1.4 ± 0.01 ^a^	0.5 ± 0.01 ^b^	0.3 ± 0.01 ^c^
MRI_280_	58.0 ± 2.2 ^c^	190.0 ± 6.0 ^a^	116.0 ± 3.1 ^b^
MRI_360_	51.0 ± 1.3 ^b^	61.0 ± 0.9 ^a^	41.0 ± 1.1 ^c^
MRI_420_	2.0 ± 0.0 ^c^	9.0 ± 0.1 ^a^	8.0 ± 0.0 ^b^
	Coffee from Oaxaca		
Moisture (%)	9.2 ± 0.02 ^a^	5.0 ± 0.05 ^b^	2.8 ± 0.03 ^c^
a*	1.4 ± 0.02 ^c^	5.3 ± 0.09 ^b^	5.3 ± 0.09 ^a^
b*	11.6 ± 0.20 ^a^	4.2 ± 0.11 ^b^	1.1 ± 0.04 ^c^
L*	53.0 ± 0.26 ^a^	25.2 ± 0.12 ^b^	25.2 ± 0.12 ^b^
Hue angle	1.4 ± 0.01 ^c^	0.66 ± 0.01 ^b^	0.27 ± 0.1 ^a^
MRI_280_	65.0 ± 2.0 ^c^	145.0 ± 7.0 ^a^	110.0 ± 5.6 ^b^
MRI_360_	55 ± 1.0 ^a^	50.0 ± 0.5 ^b^	40.0 ± 2.1 ^c^
MRI_420_	2.0 ± 0.1 ^c^	5.0 ± 0.0 ^b^	9.0 ± 0.1 ^a^
	Coffee from Hidalgo		
Moisture (%)	8.9 ± 0.03 ^a^	6.8 ± 0.01 ^b^	4.3 ± 0.12 ^c^
a*	1.6 ± 0.03 ^c^	6.1 ± 0.04 ^a^	4.7 ± 0.06 ^b^
b*	12.3 ± 0.17 ^a^	3.5 ± 0.07 ^b^	1.0 ± 0.08 ^c^
L*	54.3 ±0.26 ^a^	23.4 ±0.03 ^b^	21.8 ± 0.08 ^b^
Hue angle	1.4 ± 0.01 ^a^	0.5 ± 0.01 ^b^	0.2 ± 0.01 ^c^
MRI_280_	65.0 ± 1.8 ^c^	117.0 ± 0.6 ^b^	167.0 ± 0.1 ^a^
MRI_360_	52.0 ± 3.2 ^a^	51.0 ± 0.4 ^a^	48.0 ± 0.1 ^b^
MRI_420_	2.0 ± 0.1 ^c^	7.0 ± 0.1 ^b^	9.0 ± 0.0 ^a^

Data are mean ± standard error. Between rows, different letters indicate a significant difference between study groups, determined by Tukey’s test (*p* < 0.05). Unroasted (UN), medium roasted (MR), and high roasted (HR) coffee brew samples.

**Table 2:** Phenolic profile of coffee from four Mexican regions.

No.	Compound	Retention Time (min)	Molecular Weight	Main Transition (*m/z*)	λ Max (nm)	UN (µg/g)	MR (µg/g)	HR (µg/g)
Coffee from Chiapas								
1.	Gallic acid	1.08	169	79 > 125	270	64.5 ± 13.1 ^a^	55.1 ± 10.2 ^a^	Traces
2.	Chlorogenic acid	3.42	353	191 > 85	320	231,990 ± 5032 ^a^	13,545.2 ± 482 ^b^	10,977.2 ± 52.6 ^c^
3.	Caffeic acid	3.83	179	135 > 89	320	1125.9 ± 19.5 ^a^	63.9 ± 3.1 ^b^	26.8 ± 0.4 ^c^
4.	3,4-Dicaffecoylquinic acid	4.21	516	135 > 89	320	22,953.6 ± 78.5 ^a^	114.5 ± 14.7 ^b^	Traces
5.	Quercetin	4.23	480	479 > 303	360	48,715 ± 836 ^a^	2289.9 ± 487.4 ^b^	346.5 ± 93.7 ^c^
6.	2,4,6-trihydroxybenzoic acid	4.69	170	137 > 137	270	35.3 ± 0.2 ^a^	40.2 ± 3.1 ^a^	22.1 ± 1.7 ^b^
7.	Coumaric acid	4.90	193	178 > 134	320	315.0 ± 10.2 ^a^	Traces	Traces
8.	Ferulic acid	5.50	193	178 > 134	320	1230.7 ± 58.8 ^a^	462.71 ± 43.3 ^b^	Traces
9.	Quercetin glucuronide	6.45	480	479 > 303	360	5128.0 ± 548.2 ^a^	2077.9 ± 198.6 ^b^	892.9 ± 80.4 ^c^
10.	t-cinnamic acid	8.39	148	148 > 149	320	235.5 ± 2.2 ^a^	148.0 ± 0.4 ^b^	159.0 ± 1.7 ^b^
Coffee from Colima								
1.	Gallic acid	1.08	169	79 > 125	270	38.1 ± 1.4 ^a^	22.8 ± 6.3 ^a^	Traces
2.	Chlorogenic acid	3.42	353	191 > 85	320	158,675.8 ± 790 ^a^	30,721 ± 633.8 ^b^	19,054 ± 321.8 ^c^
3.	Caffeic acid	3.83	179	135 > 89	320	111.4 ± 10.4 ^a^	57.1 ± 3.1 ^b^	54.9 ± 0.4 ^b^
4.	3,4-Dicaffecoylquinic acid	4.21	516	135 > 89	320	12,549.9 ± 157.9 ^a^	53.9 ± 0.2 ^b^	21.6 ± 5.4 ^c^
5.	Quercetin	4.23	480	479 > 303	360	422.1 ± 9.9 ^a^	397.1 ± 18.63 ^a^	215.8 ± 2.0 ^b^
6.	2,4,6-trihydroxybenzoic acid	4.69	170	137 > 137	270	72.1 ± 1.5 ^a^	44.7 ± 0.2 ^b^	Traces
7.	Coumaric acid	4.90	193	178 > 134	320	70.3 ± 1.0 ^a^	43.8 ± 1.9 ^b^	Traces
8.	Ferulic acid	5.50	193	178 > 134	320	1973.8 ± 213.7 ^a^	178.1 ± 0.6 ^b^	13.2 ± 0.1 ^c^
9.	Quercetin glucuronide	6.45	480	479 > 303	360	2272.1 ± 49.4 ^a^	1861.3 ± 47.4 ^b^	1817.9 ± 55.8 ^b^
10.	t-cinnamic acid	8.39	148	148 > 149	320	309.5 ± 0.2 ^a^	237.02 ± 5.7 ^b^	Traces
Coffee from Oaxaca								
1.	Gallic acid	1.08	169	79 > 125	270	49.4 ± 4.9 ^a^	20.9 ± 0.9 ^b^	25.3 ± 1.4 ^b^
2.	Chlorogenic acid	3.42	353	191 > 85	320	110,882 ± 2814 ^a^	70,133 ± 4441 ^b^	11,242 ± 556.6 ^c^
3.	Caffeic acid	3.83	179	135 > 89	320	78.8 ± 9.1 ^a^	19.0 ± 1.0 ^b^	22.3 ± 0.3 ^b^
4.	3,4-Dicaffecoylquinic acid	4.21	516	135 > 89	320	5966.8 ± 241.4 ^a^	215.4 ± 16.8 ^b^	Traces
5.	Quercetin	4.23	480	479 > 303	360	56,290.6 ± 2987 ^a^	509.1 ± 16.8 ^b^	Traces
6.	2,4,6-trihydroxybenzoic acid	4.69	170	137 > 137	270	42.1 ± 3.9 ^a^	11.3 ± 0.5 ^b^	Traces
7.	Coumaric acid	4.90	193	178 > 134	320	27.7 ± 3.5 ^a^	4.9 ± 0.1 ^b^	Traces
8.	Ferulic acid	5.50	193	178 > 134	320	1371.6 ± 168.6 ^a^	719.5 ± 51.2 ^b^	46.1 ± 4.6 ^c^
9.	Quercetin glucuronide	6.45	480	479 > 303	360	5128.0 ± 548.2 ^a^	1670.6 ± 58.7 ^b^	1551.7 ± 9.3 ^b^
10.	t-cinnamic acid	8.39	148	148 > 149	320	164.3 ± 19.4 ^a^	92.3 ± 2.8 ^b^	Traces
Coffee from Hidalgo								
1.	Gallic acid	1.08	169	79 > 125	270	57.2 ± 3.1 ^a^	36.7 ± 6.1 ^b^	29.9 ± 0.1 ^b^
2.	Chlorogenic acid	3.42	353	191 > 85	320	233,296 ± 1031 ^a^	39,374.0 ± 21.6 ^b^	3295.6 ± 20.3 ^c^
3.	Caffeic acid	3.83	179	135 > 89	320	333.5 ± 43.9 ^a^	59.1 ± 1.9 ^b^	8.2 ± 0.2 ^c^
4.	3,4-Dicaffecoylquinic acid	4.21	516	135 > 89	320	30,509.4 ± 312.4 ^a^	Traces	Traces
5.	Quercetin	4.23	480	479 > 303	360	8385.6 ± 1480 ^a^	996.6 ± 60.7 ^b^	649.0 ± 5.3 ^c^
6.	2,4,6-trihydroxybenzoic acid	4.69	170	137 > 137	270	Traces	Traces	Traces
7.	Coumaric acid	4.90	193	178 > 134	320	191.6 ± 1.4 ^a^	5.5 ± 0.1 ^b^	2.9 ± 0.1 ^b^
8.	Ferulic acid	5.50	193	178 > 134	320	1658.2 ± 168.0 ^a^	1426.3 ± 3.7 ^a^	Traces
9.	Quercetin glucuronide	6.45	480	479 > 303	360	3383.7 ± 208.7 ^a^	3483.7 ± 178.6 ^a^	1987.7 ± 38.1 ^b^
10.	t-cinnamic acid	8.39	148	148 > 149	320	173.1 ± 12.5 ^a^	192.4 ± 10.9 ^a^	44.0 ± 5.6 ^b^

Compounds identified based on a fragmentation pattern by MS/MS and quantified with the use of the adjustment lines obtained with structurally similar standards. Each polyphenol abundance was expressed in µg/g of lyophilized coffee. All results are expressed as mean ± standard error. Statistical analyses were performed by ANOVA, with a Tukey value of *p* < 0.05 being statistically significant. Unroasted (UN), medium-roasted (MR), and high-roasted (HR) coffee brew samples. Different letters in the lines indicate statistically significant differences among the roasting degrees of coffee samples (*p* < 0.05; ANOVA with Tukey’s post hoc test).

**Table 3:** Digestive enzyme inhibition expressed as median inhibitory concentration (IC_50_, μg/μL) in coffee from four Mexican regions.

	Acarbose	Orlistat	UN	MR	HR
			Coffee from Chiapas		
Lipase	--	65.0 ± 2.4 ^c^	338.0 ± 3.1 ^b^	344.5 ± 3.0 ^b^	385.4 ± 4.9 ^a^
α-amylase	752.0 ± 4.9 ^d^	--	9017.0 ± 0.4 ^c^	19,853.0 ± 1.3 ^a^	18,041.0 ± 1.0 ^b^
α-glucosidase	56.2 ± 6.8 ^d^	--	5843.0 ± 1.1 ^a^	95.6 ± 3.1 ^c^	173.6 ± 2.3 ^b^
			Coffee from Colima		
Lipase	--	65.0 ± 2.4 ^b^	309.6 ± 3.8 ^a^	307.2 ± 3.7 ^a^	315.0 ± 5.3 ^a^
α-amylase	752.0 ± 4.9 ^d^	--	80,634.0 ± 2.8 ^a^	29,751.0 ±1.0 ^c^	60,778.0 ± 2.9 ^b^
α-glucosidase	56.2 ± 6.8 ^c^	--	19,108.0 ± 0.7 ^a^	111.5 ± 2.3 ^b^	121.9 ± 3.3 ^b^
			Coffee from Oaxaca		
Lipase	--	65.0 ± 2.4 ^c^	354.9 ± 2.4 ^a^	363.3 ± 2.2 ^a^	170.8 ± 4 ^b^
α-amylase	752.0 ± 4.9 ^d^	--	93,417 ± 1.5 ^a^	53,844.0 ± 0.9 ^b^	6930.0 ± 2.8 ^c^
α-glucosidase	56.2 ± 6.8 ^d^	--	3155.0 ± 1.8 ^a^	99.5 ± 4.1 ^c^	131.6 ± 2.3 ^b^
			Coffee from Hidalgo		
Lipase	--	65.0 ± 2.4 ^c^	203.8 ± 5.0 ^b^	258.8 ± 3.4 ^a^	197.7 ± 4.8 ^b^
α-amylase	752.0 ± 4.9 ^d^	--	59,707.0 ± 1.3 ^b^	22,321.0 ± 2.0 ^c^	84,878.0 ± 0.8 ^a^
α-glucosidase	56.2 ± 6.8 ^c^	--	6761.0 ± 1.6 ^a^	112.5 ± 5.9 ^b^	119.1 ± 2.1 ^b^

Values are mean ± standard error. Different letters within rows denote significant differences among groups (Tukey’s test, *p* < 0.05). Unroasted (UN), medium-roasted (MR), and high-roasted (HR) coffee brew samples.

## Data Availability

Due to ethical restrictions, the data presented in this study are available on request from the corresponding author.

## Supplementary Materials

The following supporting information can be downloaded at: https://www.mdpi.com/article/10.3390/ijms262010067/s1.

## Abbreviations

The following abbreviations are used in this manuscript:

UN	Unroasted;
MR	Medium roasted;
HR	High roasted;
MRI	Maillard reaction index;
OSTT	Oral Starch Tolerance Test;
OLTT	Oral Glucose Tolerance Test.
