# Supplementary material for: Influence of Coffee Roasting Degree from Four Mexican Regions on In Vitro Antioxidant Activity and Digestive Enzyme Inhibition and Its In Vivo Effects on Carbohydrate and Lipid Absorption
Source: Int J Mol Sci. 2025 Oct 16;26(20):10067. doi: 10.3390/ijms262010067 (PMC12564270; doi:10.3390/ijms262010067)
Supplement: Supplementary file 1 [file ijms-26-10067-s001.zip › ijms-3885704-supplementary.pdf]

Supplemental Table S1. Roast-dependent changes in active phenolics and acute bioactivity by coffee origin

Region	Roasting	CGA (µg/g)	Quercetin (µg/g)	TG (mg/dL)	Glucose (mg/dL)
CH	HR	10,977±53	346±94	145.8±35.3	107.6±10.9
CH	MR	13,545±482	2,290±487	215.3±64.6	103.7±9.7
CH	UN	231,990±5,032	48,715±836	162.9±21.5	105.2±7.4
P value		<0.0001[a, b]	<0.0001[a b, c]	0.055	0.75
CO	HR	19,054±322	216±2	178.5±47.1	97±8.8
CO	MR	30,721±634	397±19	184.4±95.9	102.3±10
CO	UN	158,675±790	422±10	91±30.2	100.6±5.3
P value		<0.0001[a, b, c]	<0.0001 [b, c]	0.02[a]	0.50
H	HR	3,296±20	649±5	125±44.3	113±12.4
H	MR	39,374±22	996.6±61	169.9±36.5	103±5.2
H	UN	233,296±1,031	8,386±1480	102.8±42.7	96±8.6
P value		<0.0001[a, b, c]	<0.0001[a, b]	0.01[a]	0.35
O	HR	11,243±557	0	147.6±23.5	97.3±6.1
O	MR	70,133±4,441	509±17	104.6±28.7	95.3±8.1
O	UN	110,882±2814	56,291±2,987	149.1±51.1	101± 7
P value		<0.0001[a, b, c]	<0.0001[a]	0.04[a, c]	0.33

Values are mean ± SD. P value was estimated using One-way ANOVA with Turkey post-hoc test.
UN, Unroasted; MR, Medium roasted; HR, High roasted
CH, Chiapas; CO, Colima; H, Hidalgo; O, Oaxaca. TG, Triglycerides at 6 h; Glucose, Glucose at 120 min.
a, p<0.01, between UN and MR coffee
b, p<0.005 between UN and HR coffee
c, p<0.01 between MR and HR

Supplemental Table S2. Roast-dependent changes on active phenolics compounds and its acute bioactivity on triglicerides and glucose levels

Roast level	Region	CGA (µg/g)	Quercetin (µg/g)	TG (mg/dL)	Glucose (mg/dL)
UN	CH	231,990±5032	48,715±836	162.9±21.5	105.2±7.4
UN	CO	158,676±790	422.1±10	91.0±30.2	100.6±5.3
UN	H	233,296±1,031	8,386±1,480	102.8±42.7	96.0±8.6
UN	O	110,882±2,814	56,291±2,987	149.1±51.1	101.0±7.0
P value		<0.0001[a, c, d, e, f]	<0.0001 [a, b, c, d, e, f]	0.003 [a, b, e]	0.15
MR	CH	13,545±482	2,290±487	215.3±64.6	103.7±9.7
MR	CO	30,721±634	397±19	184.4±95.9	102.3±10.5
MR	H	39,374±22	997±61	169.9±36.5	97.3±5.2
MR	O	70,133±4,441	509±17	104.6±28.7	95.3±8.1
P value		<0.0001[a, b, c, d, e, f]	<0.0001 [a, b, c]	0.01 [c]	0.23
HR	CH	10,977±53	346±94	145.8±35.3	107.6±10.9
HR	CO	19,054±322	216±2	178.5±47.1	97±8.8
HR	H	3,296±20	649±5	125.0±44.3	103.0±12.4
HR	O	11,242±557	0.0	147.6±23.5	97.3±6.1
HR	O	11,242±557	0.0	147.6±23.5	97.3±6.1
P value		<0.0001[a, b, d, e, f]	<0.0001 [a, b, c, d, e, f]	0.18	0.17

Values are mean ± SD. One-way ANOVA with Turkey post-hoc test.
CGA, Chlorogenic acid; UN, Unroasted; MR, Medium roasted; HR, High roasted
CH, Chiapas; CO, Colima; H, Hidalgo; O, Oaxaca. TG, Triglycerides at 6 h; Glucose, Glucose at 120 min.
a, $p<0.03$, between CH and CO coffee; b, $p<0.03$, between CH and H coffe; c, $p<0.01$ between CH and O coffe; d, $p<0.002$ between CO and O coffe; e, $p<0.04$ between CO and H coffef, $p<0.001$ between H and O coffe
